# Data matrix of site-specific environmental variables: Phytomanagement of a contaminated brownfield site

**DOI:** 10.1016/j.dib.2019.103957

**Published:** 2019-05-02

**Authors:** Carmine Guarino, Daniela Zuzolo, Mario Marziano, Domenico Cicchella, Rosaria Sciarrillo

**Affiliations:** Department of Science and Technology, University of Sannio, Italy

**Keywords:** Data matrix, Plant uptake, Trace elements, Contaminated soil, Brownfield

## Abstract

This article offers statistical analyses of trace elements (TEs) in soils and plants through a Pearson correlation matrix. The main objectives were the assessment of soil TEs sources and the evaluation of native plant response to physical and chemical characteristics of a TEs contaminated soil. Data were collected from Bagnoli brownfield site (Southern Italy). Interpretation of the data, can be found in “Identification of native-metal tolerant plant species in-situ: environmental implications and functional traits” [1]. The correlations in the matrix are based on over 76 samples and 31 site-specific environmental variables.

**Table:** Specification table

Subject area	Environmental Sciences; geochemistry, biology.
More specific subject area	Environmental geochemistry; phytoremediation; contaminated site.
Type of data	Table, figure
How data was acquired	Pseudo-total TEs fraction was determined by ICP OES (Varian Inc., VistaMPX) in plant and rhizosphere soil. The elemental bioavailable fraction in rhizosphere soil was determined for As (NH_4_OAcetate extraction), Cd, Cu, Pb and Zn (DTPA extraction) and for Tl (NH_4_OAcetate extraction).
Data format	Analyzed
Experimental factors	In-situ native plant species and rhizosphere soils
Experimental features	The roots and leaves of each plant and rhizosphere soil samples of the above plants were collected and stored in clean polypropylene containers at each site and kept at 4 °C for further treatment and analysis.
Data source location	Bagnoli brownfield site, Southern Italy (between 40°49′30″ 91 and 40°47′30″Nord, and 14°9′30″ and 14°12′0″ East).
Data accessibility	Data are accessible within this article
Related research article	[1] Guarino, C., Zuzolo, D., Marziano, M., Baiamonte, G., Morra, L., Benotti, D., Gresia, D., Robortella Stacul, E., Cicchella, D., Sciarrillo, R., 2019. Identification of native-metal tolerant plant species in situ: environmental implications and functional traits. Science of the Total Environment 650, 3156–3167. https://doi.org/10.1016/j.scitotenv.2018.09.343

**Table:** 

**Value of the data** • Correlation data analyses allow to distinguish anthropogenic sourced metals from natural origin ones. • Information gained about variables correlation will enrich the development of further experiments. • Pearson correlation matrix that were used to generate data can be of use for further research by other who have interest in understanding the phytomanagement of a contaminated brownfield site on a broader scale.

## 1 Data

**Fig. 1:**
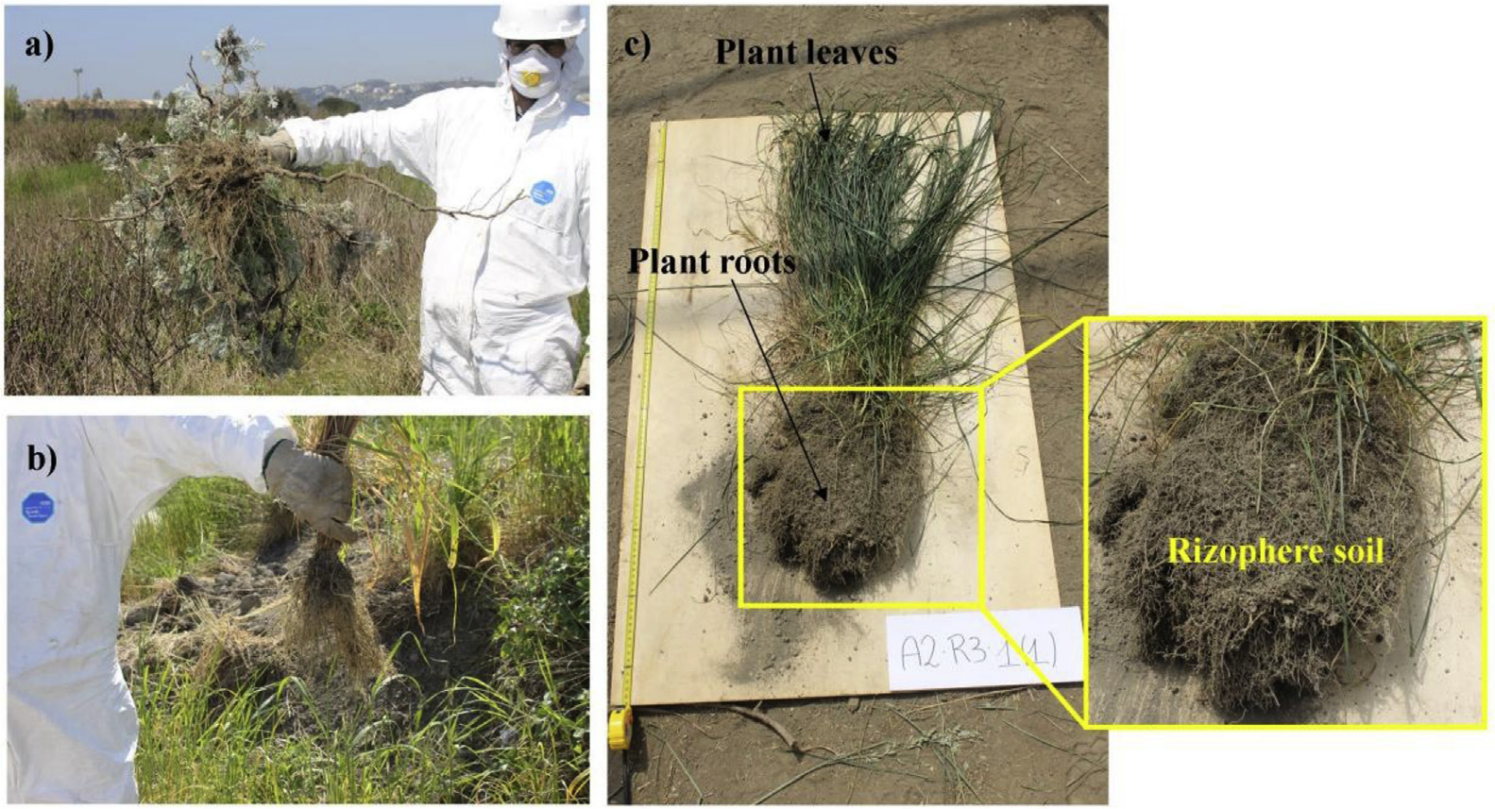
a), b) Sampling activity of native plant species from Bagnoli brownfield site (Southern Italy); c) sampled media from the study area: plant roots, plant leaves and rhizosphere soil of native *Festuca arundinacea* species.

The data derive from an investigation undertaken to screen suitable native plants for the phytoremediation of the second largest integrated steelworks in Italy (Bagnoli brownfield site) (Fig. 1 a, b). Here, a correlation matrix contain relationship estimation between thirty-one variables is presented. Variables include TEs content in rhizosphere soils, parameters of agronomic interest and TEs content in roots and leaves of the analyzed plant species. The matrix is based on seventy-six samples. Data below detection limit (IDL) were assigned 50% of IDL. The ** marked correlations are significant at p < 0.010; the* marked correlations are significant at p < 0.05.

## 2 Experimental design, materials and methods

### 2.1 Experimental design

The study area is Bagnoli brownfield site, which soils are characterized by the presence of a cover made up of waste produced inside the industrial area (furnace scum and slag) mixed with pyroclastic terrain [2]. These features lead to a soil multielement contamination. Field-survey aimed to assess the potential of native plant for phytomanaging this site. Seventy-six sampling sites were selected based on the presence of plant colonists and vegetation assemblage patterns and the dominant plant species at each sampling site were identified. Plants were sampled into two sections (roots and leaves - Fig. 1c) and stored in clean polypropylene containers. Rhizosphere soil samples of the above plants were also collected by shaking the roots, stored in labelled polypropylene containers, and kept at 4 °C for further treatment and analysis.

### 2.2 Sample preparation and analyses

Plant samples were thoroughly washed with tap water to remove dust particle sand and carefully washed with deionized water. Following, they were acid-digested according to the USEPA method [3], [4] and analyzed for TEs content by an ICP-OES (Varian Inc., Vista MPX).

All soil samples were air-dried to avoid Hg volatilization and sieved at the <2 mm fraction to collect 30 g for chemical analysis. Furthermore, the following parameters of agronomic interest were determined: pH, EC at 25 °C, CEC, total organic Carbon, total N, Ca, Mg, Na, P and K. Then soil samples were oven-dried at 105 °C for 24 hours and nitric acid-digested in a microwave oven (CEM, MARSXpress) according to the USEPA method, (USEPA, 1995a,b). After mineralization, soil extracts were filtered (0.45 μm PTFE), diluted and analyzed. Total content of As, Cd, Cu, Pb, Tl and Zn in soils extracts were determined by an ICP-OES (Varian Inc., VistaMPX).

The elemental bioavailable fraction in rhizosphere soil was determined according to (i) [5] for As (NH_4_OAcetate extraction), (ii) [6] for Cd, Cu, Pb and Zn (DTPA extraction) and (iii) [7] for Tl (NH_4_OAcetate extraction).

### 2.3 Statistical data analyses

Some geochemical variables violated the normality assumption. Correlation analyses among the variables were determined in order to assess the kind of relationships existing among the analyzed variables using Pearson both on raw (Supplementary data 1) and on log-transformed data (Supplementary data 2).

The bivariate analysis measures the strength of association between two variables and the direction of the relationship. Variables with more than 65% of data below detection limit (IDL) were excluded from correlation analyses. The ^∗∗^ marked correlations are significant at p < 0.010; the ^∗^ marked correlations are significant at p < 0.05. Ranges between −1 and +1 quantifies the direction and strength of the linear association between the two variables. The sign of the correlation coefficient indicates the direction of the association. The magnitude of the correlation coefficient indicates the strength of the association.

## References

[1] Guarino C., Zuzolo D., Marziano M., Baiamonte G., Morra L., Benotti D., Gresia D., Robortella Stacul E., Cicchella D., Sciarrillo R. (2019). Identification of native-metal tolerant plant species *in* situ: environmental implications and functional traits. Sci. Total Environ..

[2] De Vivo B., Lima A. (2018). The Bagnoli-Napoli Brownfield Site In Italy: Before and After the Remediation. Environmental Geochemistry (second ed.): Site Characterization, Data Analysis and Case Histories.

[3] USEPA (1995). EPA Method 3051: microwave assisted acid digestion of sediments, sludges, soils, and oils. Test Methods for Evaluating Solid Waste.

[4] USEPA (1995). EPA Method 3052: microwave assisted acid digestion of siliceous and organically based matrices. Test Methods for Evaluating Solid Waste.

[5] Hudson-Edwards K.A., Houghton S.L., Osborn A. (2004). Extraction and analysis of arsenic in soils and sediments. Trends Anal. Chem..

[6] Martens D.C., Lindsay W.L., Westerman R.L. (1990). Testing soils for copper, iron, manganese, and zinc. Soil Testing and Plant Analysis.

[7] Al-Najar H., Kaschl A., Schulz R., Roemheld V. (2005). Effect of thallium fractions in the soil and pollution origins on Tl uptake by hyperaccumulator plants: a key factor for the assessment of phytoextraction. Int. J. Phytoremediation.

