# Supplementary material for: Data matrix of site-specific environmental variables: Phytomanagement of a contaminated brownfield site
Source: Data Brief. 2019 May 2;25:103957. doi: 10.1016/j.dib.2019.103957 (PMC6545384; doi:10.1016/j.dib.2019.103957)
Supplement: Supplementary file 1 — Multimedia Component 1 [file mmc1.docx]

Dear Editor in Chief

Data in Brief

Benevento, 31/01/2019

The Authors of the manuscript “Data matrix of site-specific environmental variables: TEs phytomanagement of a contaminated brownfield site” by Carmine Guarino, Daniela Zuzolo, Mario Marziano, Domenico Cicchella and Rosaria Sciarrillo declare that there is no conflict of interest.

Thank you and best regards

Rosaria Sciarrillo
